# Periportal fibrosis, liver and spleen sizes among *S. mansoni* mono or co-infected individuals with human immunodeficiency virus-1 in fishing villages along Lake Victoria shores, North-Western, Tanzania

**DOI:** 10.1186/s13071-015-0876-4

**Published:** 2015-05-07

**Authors:** Humphrey D Mazigo, David W Dunne, Domenica Morona, Therese E Lutufyo, Safari M Kinung’hi, Geofrey Kaatano, Fred Nuwaha

**Affiliations:** Department of Medical Parasitology and Entomology, School of Medicine, Catholic University of Health and Allied Sciences, P.O. Box 1464, Mwanza, Tanzania; Department of Disease Control and Environmental Health, School of Public Health, College of Health Sciences, Makerere University, P.O. Box 7072, Kampala, Uganda; National Institute for Medical Research, Mwanza Research Centre, P.O. Box 1462, Mwanza, Tanzania; Department of Pathology, Division of Microbiology & Parasitology, Cambridge University, Tennis Court Road, Cambridge, CB2 1QP UK

**Keywords:** *S. mansoni*, HIV-1, Co-infection, Periportal fibrosis, Liver and spleen sizes, Tanzania

## Abstract

**Background:**

The pathogenesis of *S. mansoni* infection involves chronic inflammatory responses to parasite eggs which can be associated with a characteristic periportal fibrosis (PPF) and the progression to severe hepatosplenic disease. The effects of HIV-1 co-infection and the influence of CD4^+^ cell numbers on these clinical manifestations of chronic *S. mansoni* are not known. To understand the effects of HIV-1 co-infection on these morbidities, we examined *S. mansoni* ultrasound-detectable morbidities in relation to HIV-1 infection and CD4^+^ cell counts, and other factors in fishing communities where the two infections are present.

**Methods:**

Ultrasonographical examination was conducted during a cross-sectional study of 1,671 (aged 21–55 years) individuals in North-Western Tanzania. Blood samples were obtained for HIV-1 screening and CD4^+^ cell quantification. A single stool sample was examined for *S. mansoni* eggs using the Kato-Katz technique. A questionnaire was used to collect socio-demographic-economic information.

**Results:**

The prevalence of PPF (grade C-F) was 13.79% and 15.01% for the HIV-1 infected and non-infected individuals (*P* = 0.72). Male gender (*P*< 0.001), age group 21–30 years (*P*< 0.028) and, residential time of 11–20 (*P*< 0.01) and ≥21 years (*P*< 0.01) were associated with PPF in *S. mansoni* infected individuals. The height-adjusted measurements of the left liver lobe were significantly larger in HIV-1/*S. mansoni* co-infected compared to *S. mansoni* only-infected individuals (t = −2.0702, *P*< 0.039). Predictors of the height-adjusted measurements of the left liver lobe and spleen were age, male gender, malaria infection, fishing occupation, village of residence and heavy intensity of *S. mansoni* infection. After accounting for these factors, neither HIV-1 infection nor CD4^+^ cell counts predicted PPF, hepatosplenomegaly, measurements of the liver or spleen. Height-adjusted ultrasound measurements of the left liver lobe did not correlate with the CD4^+^ cells counts in co-infected individuals (r = −0.16, *P* = 0.084).

**Conclusion:**

*S. mansoni*-related PPF, liver and spleen enlargement are prevalent in the study population. The intensity of *S. mansoni* infection was associated with the enlargement of liver, spleen and hepatosplenomegaly. The PPF grades observed were similar in both HIV-1/*S. mansoni* co-infected and in those only infected with *S. mansoni.* There was no evidence that HIV-1 infection or CD4^+^ cells counts were associated with these *S. mansoni* morbidities.

## Background

Chronic *S. mansoni* infection can result in severe complications and life-threatening hepatosplenic disease [1,2]. The pathogenesis of the hepatosplenic form of the disease is a result of the chronic immunological responses mediated by CD4^+^ T-lymphocytes directed against *S. mansoni* eggs which are trapped within the host body tissues including the liver [2,3]. Immunological responses against the eggs of the parasite trapped in the liver are responsible for the development of peri-ova granulomas, chronic exposure to which can be associated with the development of fibrosis of the liver portal tracts and associated morbidities [4,5]. Experimental murine model studies suggest that granuloma formation in *S. mansoni* infection is a CD4^+^ T-cells dependent process [3,5-7]. The CD4^+^ cells are predominantly associated with the secretion of Th_2_ cytokines which contributes to the development of hepatic fibrosis [8,9]. Evidence from immunodeficient mice (nude, T-cell depleted and severe combined immunodeficiency (SCID) mice characterized by the absence of CD4^+^ T-lymphocytes and egg antibodies response) [10-13], indicate that these animals suffer exacerbated severe hepatic parenchyma damage with reduced granulomatous responses [10-13]. However, there are differences between liver morbidity in *S. mansoni* infected humans and mice; for example, infected mice do not develop the PPF seen in human infections [8,14].

In human hosts, hepatosplenic disease is often accompanied by hepatic and splenic enlargement; progressive periportal fibrosis (PPF) can lead to portal hypertension and its sequelae [1-3,6,8,9], including ascites, liver surface irregularities and portal-systemic venous shunts, with the risk of oesophageal varices and haematemesis [1-3,8,9]. Many of these manifestations of chronic morbidity can be detected and measured by ultrasonography and the degree of PPF severity is classified using a recommended World Health Organization grading scale, agreed under the Niamey protocol [15-17]. Although immunological responses play a crucial role in the development of hepatosplenic disease, epidemiological [18] and demographic factors are also very important, including the duration of residence in schistosomiasis endemic areas [16,19,20], socio-economic factors such as the involvement in fishing activities [16], as well as environmental [18], genetical [21], parasitological factors [18,20-22] and co-infection with other tropical diseases such as malaria [23,24]. Hepatomegaly or splenomegaly associated with malaria infection is mainly a result of repeated inflammatory responses characterized by hyperplasia of reticuloendothelial and lymphoid tissues [25], which can be exacerbated by chronic *S. mansoni* infection [23-25]. Any study of the relationship between *S. mansoni* infection, HIV-1 infection, hepatosplenic disease and its squelae, has to take account of the effects of such factors.

The overall pathogenesis of HIV-1 infection is triggered by the destruction or depletion of CD4^+^ T-lymphocytes which consequently lead to the loss of immune competence [26,27] and an increase in the susceptibility of the infected individuals to other infectious diseases [26]. It has been hypothesized that the destructions of CD4^+^ T-lymphocytes by HIV-1 infection may alter the sizes of the left liver lobe and the patterns of hepatic fibrosis. This, in turn, may increase the risk of hepatic parenchymal damage due to an insufficient production of the Th_2_ cytokines which are responsible for fibrogenesis [28,29]. In-vitro studies have shown that T-cells from individuals co-infected with HIV-1 and *S. mansoni* produced lower IL-4 and IL-10 and IFN-γ [28]. The inability to stimulate these cells may lead to the development of a severe hepatic morbidity. Furthermore, the observation that CD4^+^ T-cells are required for *S. mansoni* eggs excretion in mice [30,31] and that HIV-1 positive Kenyans infected with *S. mansoni* have impaired parasite eggs excretion that correlates with decreased CD4^+^ cell counts [32], suggest that the effects of HIV-1 on CD4^+^ cells counts could lead to more parasites eggs being retained in the host body organs. Thus, individuals co-infected with HIV-1 and *S. mansoni* may have altered ultrasonography detectable morbidities such as severe hepatomegaly and splenomegaly compared to those infected with *S. mansoni* alone. However, to date, limited evidence is available [33].

To investigate the hypothesis that individuals co-infected with HIV-1 and *S. mansoni* suffer severe ultrasound detectable morbidities as compared to individuals infected with only *S. mansoni* infection. The present study examined the prevalence and intensities of *S. mansoni* infection and the related morbidities using ultrasonography in HIV-1 infected and un-infected population (aged 21–55 years) to define the type of morbidities and evaluate the role of various known demographic and parasitological factors in development of these *S. mansoni* morbidities and their association with left liver lobe and spleen sizes, and hepatosplenomegaly. In addition, we tested if the ultrasound measurements of the left liver lobe are associated with the level of CD4^+^ cell counts in individuals co-infected with HIV-1 and *S. mansoni* infections.

## Methods

### Study area, population and design

The study area, inclusion and exclusion criteria and sampling procedures of the study participants are described in Mazigo *et al*. [34,35]. In brief, the study included four villages of North-Western Tanzania, bordering the southern shores of the Lake Victoria. All people who had lived in the study villages for more than two years (referred to as permanent residents) and aged 21 to 55 years were eligible for enrolment. Individuals with a history of treatment for schistosomiasis (praziquantel) in the past six months and those who were on antiretroviral treatment (ART) were excluded from the study at the baseline. A two-step sampling procedure was used to select households and household members to participate in the study [34,35].

### Data collection

#### (i) Demographic information

Demographic information on sex, age, occupation, marital status, village of residence, number of years lived in current residence and level of education were collected with the use of a questionnaire [34]. The detailed demographic characteristics of the study participants are described in Mazigo *et al*. [34]. In brief, complete parasitological data and ultrasonographical data were obtained from 1,785 individuals aged 21–55 years, excluding n = 114 individuals who were positive in an hepatitis C serological assay. Therefore, a total of 1,671 individuals’ data were available for the present analysis.

#### (ii) Human Immunodeficiency Virus screening and CD4^+^ analysis

Human Immunodeficiency Virus-1 testing was conducted according to the Tanzanian National HIV algorithms which recommend the use of a rapid test qualitative immunoassay [36]; other procedures are described in detail in Mazigo *et al*. [34,35]. The quantification of CD4^+^ cells was done using a FACSCalibur machine (Becton Dickinson-BD Biosciences, San Jose, CA, USA) following standard procedures [37]. Baseline results on HIV-1 infection are described in detail in Mazigo *et al*. [34,35]. Briefly, the prevalence of HIV-1 was 6.29%, (95% CI; 3.59-11.04).

#### (iii) Parasitological screening for *Schistosoma mansoni*

A single stool sample was collected from all study participants. Four Kato Katz thick smears were prepared from different parts of the single stool sample using a template of 41.7 mg (Vestergaard Frandsen, Lausanne, Switzerland), following a standard protocol [38-40]. After 24 hours, the smears were independently examined for *S. mansoni* eggs by two experienced laboratory technicians of the National Institute for Medical Research (NIMR) laboratory. For quality assurance, a random sample of 10% of the negative and positive Kato Katz thick smears were re-examined by a third technician. At baseline, the overall prevalence of *S. mansoni*, HIV-1 infections and their relationship with demographic characteristics of the study participants are described in details in Mazigo *et al*. [34]. Briefly, the overall prevalence of *S. mansoni* was 47.85%, (95% CI; 40.46-56.57). Only 40% (n = 50) of the HIV-1 infected individuals were co-infected with *S. mansoni* [34]*.*

#### (iv) Parasitological screening for malaria

A finger prick blood sample was used to prepare thick and thin blood films for malaria parasites diagnosis and stained in 10% Giemsa (Sigma). The slides were examined under a microscope using objective X 100 under oil-immersion [39]. *Plasmodium falciparum* parasite density was determined, as described elsewhere [39]. At baseline screening, the overall prevalence of malaria infection was 8.02% (134/1,671) and the overall geometrical mean parasite density was 450.41parasites/μL. The prevalence of malaria did not vary by sex (χ^2^ = 0.0425, *P* = 0.84) and age of the study participants (χ^2^ = 5.7853, *P* = 0.12), though the age group 21–30 years (11.39%) had a higher prevalence compared to other age groups. In terms of occupation, those involved in farming had a higher prevalence (10.8%), than those involved in other economic activities but the difference was not significant (*P* = 0.09).

#### (iv) Anthropometric measurements

Body height was measured by means of a locally constructed stadiometer using a tape measure fixed on a wooden board, whereas body weight was measured using electronic bathroom scales.

#### (v) Clinical and ultrasound examination

All study participants were clinically examined for consistency of liver and spleen after submitting a single stool sample. The procedures used for examination are described in detail by Vennervald *et al.* [41]. An ultrasonographical examination of the study participant was carried out by two experienced radiographer using a portable ultrasound machine (Aloka, Tokyo). The modified Niamey Protocol was applied for classification of the level of pathology [15]. Both the examiner and the assistant were blinded with respect to the HIV-1 serostatus and *S. mansoni* results. The liver texture patterns, peripheral portal branches (PPBs), periportal fibrosis (PPF), thickness of PPB walls, spleen size, splenic vein (SV) diameter and ascites were assessed. Periportal fibrosis (PPF) was defined according to WHO [15] and the degree of PPF was categorized as A, B, C, D, E and F [15]. Periportal fibrosis grade A and B were classified as normal.

The liver length was measured along the para-sternal line (PLL), whereas the spleen length was measured along the mid clavicular line (MCL). The measurement of portal vein diameter (PVD) was done at a midway between its entrance of the portal hepatica and its bifurcation inside the liver [15].

### Treatment

All individuals found infected with *S. mansoni* at baseline (aged 21–55 years), irrespective of their HIV-1 sero-status or CD4^+^ cell count levels, were treated with a single dose of praziquantel (40 mg/kg). Treatment was performed under direct observation (DOT) of a qualified nurse. Study participants received a cup of tea and some bread before treatment to relieve the mild side effects of praziquantel. In addition, after treatment, participants were requested to remain at the treatment point for two hours to observe and manage any possible adverse effects.

### Ethical considerations

Ethical approval was obtained from the Higher Degrees Research and Ethics Committee of the School of Public Health, Makerere University (Institutional Review Board (IRB) -00005856/2011) and from the Bugando University College of Health Sciences and Allied Sciences-Institutional Review Board, (BREC/001/32/2011). Ethical clearance was granted by the National Ethical Review Committee, National Institute for Medical Research, Tanzania and the study was registered in the clinical trial network, Clinical Trial (Number:- NCT-01541631). The study received authorization from the regional and district administrative authorities of Mwanza region and Ilemela district. Swahili translated informed assent and consent forms were used to obtain children (15- <18 years) and adult participants’ consent respectively. For illiterate individuals, a thumb print was used to sign the assent and consent forms after a clear description of the study objective. All HIV-1 infected individuals found to have CD4^+^< 350 cells/μL were referred to the Care and Treatment Clinic (CTC) for assessment of their eligibility for antiretroviral therapy (ART).

### Data management and analysis

The data were double entered using CSPro and the final data set was stored in a MySQL database at the National Institute of Medical Research (NIMR) Mwanza centre. Data were checked for consistency and errors were cleaned. Data analysis was performed using stata version 12 (Stata Corp, College station, Texas, USA). The ultrasound measurements of the liver, spleen and portal vein diameter of the study participants were compared between those who did or did not have detectable *S. mansoni* infection using t-test. Similarly, the t-test was used to compare organs sizes between individuals who were mono-infected with *S. mansoni* and those who were co-infected with *S. mansoni* and HIV-1.

To define the cut-off point between normal and enlarged organs for ultrasound measurements, such as enlarged spleen, enlargement of the left liver lobe and portal vein dilatation, the Niamey protocol was used [15].

To assess the contributions of intensities of *S. mansoni* and HIV-1 infections on the extent of the liver and spleen enlargement, linear regression models were constructed for height-adjusted measurements of the left liver lobe and the spleen. Explanatory variables included in the models were sex, age, occupation, village of residences, malaria infection, HIV-1 serostatus and intensities of *S. mansoni*. Bivariate and multivariable logistic regression models were also constructed to identify factors associated with PPF and hepatosplenomegaly. At bivariate analysis, individuals factors were analyzed with the outcome of interest and explanatory variable with *P*-value <0.2 were considered for multivariable analysis. Explanatory variables entered were the same for linear regression. Unadjusted and adjusted Odd Ratio and their 95% confidence interval (95% CI) were generated and used to measure the strength of association between outcome and explanatory variables. The Spearman correlation test was used to test any correlation between height adjusted left liver lobe ultrasound measurements and CD4^+^ counts in individuals co-infected with HIV-1 and *S. mansoni.* Values for *P*< 0.05 were considered significant.

## Results

### (a) Prevalence of periportal fibrosis (PPF) and associated factors

The overall prevalence PPF (grades C-F) was 14.78% (247/1,671, 95% CI: 13.28-16.72). In relation to *S. mansoni* infection, of the individuals detected with PPF, 52.23% (129/247) had detectable *S. mansoni* eggs in their stool samples and 47.77% (118/247) had no detectable *S. mansoni* eggs. In general, the prevalence of PPF did not vary by *S. mansoni* infection status (χ^2^ = 2.6768, *P* = 0.10) and categories of intensities of infection in terms of egg count (epg) (χ^2^ = 6.1379, *P* = 0.11). The stratification of PPF grades and infection with *S. mansoni* are shown Table 1 and there was no significance difference between individuals who were either infected or not with *S. mansoni* infection (*P* = 0.39). Of the 247 individuals with PPF, 29.55% (73/247) were grade C, 52.63% (130/247) D, 14.17% (35/247) E and 3.64% (9/247) were grade F. Of the individuals infected with *S. mansoni* and having PPF, 31.78% (n = 41/129) in grade C, 49.61% (n = 63/129) in grade D, 12.02% (n = 20/129) in grade E and 3.10% (n = 4/129) in grade F had egg positives Kato Katz slides.

**Table 1:** **Prevalence of peri-portal grades and hepatosplenomegaly in relation to** ***Schistosoma mansoni*** **infection status**

**Variables**	***Schistosoma mansoni*** **status**		÷^**2**^	***P*** **-values**
	**Negative (N = 831)**	**Positive (N = 840)**		
**Hepatosplenomegaly**				
Both liver + spleen not enlarged	305	291	4.5425	0.21
	(36.70)	(34.64)		
Only spleen enlarged	78	91		
	(9.39)	(10.83)		
Only liver enlarged	327	313		
	(39.35)	(37.26)		
Both spleen and liver enlarged	121	145		
	(14.56)	(17.26)		
**Peri portal fibrosis grades**				
Normal	713	710	4.1566	0.39
	(85.80)	(84.52)		
Grade C	32	41		
	(3.85)	(4.88)		
Grade D	66	64		
	(7.94)	(7.62)		
Grade E	15	20		
	(1.81)	(2.38)		
Grade F	5	4		
	(0.60)	(0.48)		

In relation to other demographic factors, males individuals had a higher prevalence of PPF than females (157/247, 20.08% *versus* 90/247, 10.31%, χ^2^ = 30.9943, *P*< 0.001) and a higher prevalence of more severe fibrosis (grade D, E, or F) (*P*< 0.001). PPF prevalence did not significantly vary by age group (χ^2^ = 1.3521, *P* = 0.72), although the age group 31–40 years (15.08%) and 41–50 years (16.94%) groups had the highest PPF prevalence. Individuals involved in fishing activities had the highest prevalence of PPF (25.30%, χ^2^ = 26.0954, *P*< O.001). The prevalence of PPF varied significantly by villages of residence with Kayenze (17.02%) and Igalagala (19.65%) having the highest prevalence (χ^2^ = 16.5998, *P*< 0.001) and duration of residence (χ^2^ = 14.2821, *P* = 0.01), with those reported living in the study for 11–20 years (19.32%) and ≥ 21 years (18.16%) having the highest prevalence.

In relation to HIV-1 infection, 13.79% and 15.01% of the HIV-1 infected and uninfected individuals had PPF (χ^2^ = 0.1258, *P* = 0.72) respectively. Of these n = 4 had grade C, n = 8 had grade D and n = 4 had grade E-F. The prevalence of PPF did not differ by HIV-1 serostatus, (χ^2^ = 0.1258, *P* = 0.72).

To ascertain the importance of HIV-1 infection as predictor of PPF, a logistic regression model that included *S. mansoni* infection or number of *S. mansoni* eggs per gram of faeces, duration of residency, age, village of residence, occupation, malaria infection and sex as explanatory factors was developed among *S. mansoni* infected individuals with PPF as the outcome variable (Table 2). A bivariate analysis, male gender (*P*< 0.004) and residential year of 11–20 years (*P*< 0.035) were associated with PPF. At multivariable analysis, male gender (AOR = 2.27, 95% CI; 1.41 – 3.65, *P*< 0.001), age group 21–30 years (AOR = 2.45,95% CI: 1.09 - 5.47, *P*< 0.028) and residential time, 11–20 years (AOR = 3.52,95% CI; 1.38-8.93, *P*< 0.01) and ≥21 years (AOR = 2.66,95% CI: 1.27-5.58, *P*< 0.01) (Table 2).

**Table 2:** **Factors associated with Periportal fibrosis among resident of fishing villages of North-western Tanzania**

**Variable**	**cOR**	**95% CI**	***P*** **-value**	**aOR**	**95% CI**	***P*** **-value**
**Sex**						
Female	1			1		
Male	1.79	1.21 – 2.68	0.004	2.27	1.41 – 3.65	0.001**
**Age (in years)**						
21 – 30	1.35	0.71 – 2.53	0.36	2.45	1.09 – 5.47	0.028**
31 – 40	1.32	0.68 – 2.54	0.40	1.42	0.62 – 3.26	0.40
41- 50	1.16	0.53 – 2.55	0.71	1.34	0.56 – 3.28	0.51
51 - 55	1			1		
**Occupation**						
SSB*	1			1		
Farming	0.74	0.41 – 1.34	0.33	------	------	-------
Fishing	1.26	0.64 – 2.45	0.50	------	------	-------
**Village of residence**						
Igombe	1			1		
Sangabuye	0.52	0.26 – 1.03	0.06	0.39	0.18 – 0.85	0.018
Kayenze	1.17	0.71 – 1.94	0.53	1.05	0.59 – 1.85	0.19
Igalagala	1.44	0.79 – 2.62	0.23	1.74	0.74 – 4.06	0.19
**Residential years**						
≥ 2 - 5	1			1		
6 – 10	1.18	0.57 – 2.44	0.64	1.09	0.49 – 2.45	0.81
11 – 20	2.02	1.05 – 3.87	0.035	3.52	1.38 – 8.93	0.01**
≥ 21	1.42	0.79 – 2.57	0.24	2.66	1.27 – 5.58	0.01**
**Malaria infection**						
No	1			1		
Yes	0.57	0.26 – 1.24	0.16	0.72	0.32 – 1.61	0.43
HIV-1 infection						
No	1					
Yes	0.90	0.39 – 2.08	0.81	-----	-----	-----
**Intensity of** ***S. mansoni i*** **infection (epg)**						
1-100	1			1		
101 – 399	0.91	0.55 – 1.49	0.72	0.84	0.47 – 1.46	0.53
≥400	1.38	0.89 – 2.13	0.14	1.11	0.66 – 1.86	0.68

SSB* = small scale business, cOR = Crude Odd Ratio, aOR = Adjusted Odd Ratio.

**significant *P*-values for factors associated with peri-portal fibrosis.

### (b) Comparison of the height adjusted mean deviations of organs in relation to infection with *S. mansoni*

Table 3 shows the comparison of mean deviations of organs in relation to *S. mansoni* infection. In general, the height-adjusted ultrasound measurements of the left liver lobe (t = 0.8851, *P* = 0.38), spleen (t = −0.5639, *P* = 0.57) and portal vein diameter (t = −0.1710, *P* = 0.86) did not differ significantly among individuals either infected or not with *S. mansoni* infection.

**Table 3:** **Comparison of the height adjusted mean deviations of organs stratified by infection with** ***S. mansoni***

**Organomegaly**	***Schistosoma mansoni*** **status**		***F*** **-ratio**	***P*** **-values**
	**Not Infected with** ***S. mansoni*** **(N = 831) deviations from mean (95% CI)**	**Infected with** ***S. manson*** **i (N = 840) deviations from mean (95% CI)**		
**Liver**				
Left liver lobe length	2.34 (2.22 – 2.47)	2.27 (2.14 – 2.39)	0.8851	0.38
**Spleen**				
Spleen length	1.19 (1.06 – 1.31)	1.23 (1.11 – 1.35)	−0.5639	0.57
**Portal vein Diameter**				
Portal vein length	1.03 (0.96 – 1.10)	1.04 (0.97 – 1.11)	−0.1710	0.86

### (c) Comparison of the height-adjusted size of the of the organs in relation to HIV-1 infection

Table 4 shows a comparison of the height-adjusted size of the left liver lobe, spleen and portal vein diameter in relation to HIV-1 infection. The height-adjusted ultrasound measurements of the left liver lobe were significantly larger in individuals co-infected with HIV-1 and *S. mansoni* compared to *S. mansoni* only infected individuals (t = −2.0702, *P*< 0.039). However, the height-adjusted ultrasound measurements of the spleen (t = −1.3664, *P* = 0.17) and portal vein diameter (t = 0.8072, *P* = 0.42) did not differ significantly between individuals co-infected with HIV-1 and *S. mansoni* infection compared to *S. mansoni*-only infected individuals.

**Table 4:** **Comparison of the height-adjusted size of the left liver, spleen and portal vein diameter in relation to infection with HIV-1 serostatus**

**Organomegaly**	**HIV-1 serostatus**		**F-ratio**	***P*** **-values**
	**HIV-1 negative infected with** ***S. mansoni *** **(N = 790) deviations from mean (95% CI)**	**HIV-1 positive co-infected with** ***S. manson*** **i (N = 50) deviations from mean (95% CI)**		
**Liver**				
Left liver lobe length	2.23 (2.11 – 2.36)	2.79 (2.23 – 3.37)	−2.0702	0.039**
**Spleen**				
Spleen length	1.21 (1.08 – 1.34)	1.57 (1.14 – 2.00)	−1.3664	0.17
**Portal vein Diameter**				
Portal vein length	1.04 (0.97 – 1.12)	0.92 (0.69 – 1.16)	0.8072	0.42

**significant *P*-values.

### (d) Correlation between the height-adjusted ultrasound measurements of the left liver lobe, spleen, portal vein diameter and intensity of *S. mansoni* infection

The Spearman correlation test was used to determine whether the size of the left liver lobe and spleen correlated with the intensity of *S. mansoni* infection. For individuals infected with *S. mansoni*, the intensity of infection remained significantly correlated with height-adjusted length of the left liver lobe (r = 0.073, *P*< 0.0244) and height-adjusted length of the spleen (r = 0.089, *P*< 0.006). However, the height-adjusted portal vein (r = 0.004, *P* = 0.89) and portal branch wall thickness (r = 0.0656, *P* = 0.73) measurements did not correlate with *S. mansoni* intensity of infection.

### (e) Correlation between the height-adjusted ultrasound measurements of the left liver lobe and CD4^+^ cell counts

**Figure 1:**
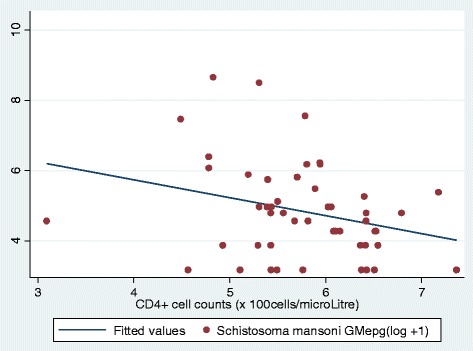
Scatter plot of correlation of left liver lobe ultrasound measurements and CD4^+^ counts in individuals co-infected with HIV-1 and *S. mansoni*. Ultrasound measurements were adjusted for height of individuals. The no correlation was observed between left liver lobe ultrasound measurements and CD4^+^ counts (r = −0.16, *P* = 0.084).

For individuals who were co-infected with HIV-1 and *S. mansoni*, the height-adjusted ultrasound measurements of the left liver lobe did not correlate with CD4^+^ T-cell counts among the co-infected individuals (r = −0.16, *P* = 0.084) Figure 1.

### (f) Predictors of left liver lobe hepatomegaly, splenomegaly and hepatosplenomegaly

To assess the contribution of HIV-1, *S. mansoni* and other explanatory variables on the extent of left liver lobe and spleen, linear regression models were developed with height adjusted ultrasound measurements of the left liver lobe and spleen as continuous outcome variables for individuals who had detectable *S. mansoni* eggs in faecal samples. The ultrasound adjusted left liver lobe measurements were significantly associated with the age of the study participants (*P*< 0.038), malaria infection (*P*< 0.038) and intensities of *S. mansoni* infection (*P*< 0.01). HIV-1 infection was not a significant predictor of left liver lobe ultrasound measurements (*P* = 0.069) (Table 5).

**Table 5:** **Predictors of left liver lobe size among residents of fishing villages of North-Western Tanzania infected with** ***S. mansoni***

**Variable**	**â**	**SE**	**95% CI**	***P*** **-values**
Sex	−0.1735	0.1390	−0.446 – 0.0994	0.21
Age	0.0100	0.0048	0.00058 – 0.0195	0.038**
**Occupation**				
SSB*	1			
Peasants	0.1357	0.2147	−0.29 – 0.55	0.53
Fishing	0.1474	0.2649	−0.37 – 0.67	0.58
**Villages of residence**				
Igombe	1			
Sangabuye	0.2185	0.2025	−0.17 – 0.62	0.28
Kayenze	0.1104	0.1762	−0.24 – 0.46	0.53
Igalagala	0.3354	0.2873	−0.23 – 0.89	0.24
**Malaria infection**				
No	1			
Yes	0.4142	0.1989	0.023 – 0.80	0.038**
**HIV-1 serostatus**				
Negative	1			
Positive	0.5258	0.2889	−0.04 – 1.093	0.069
***Schistosoma mansoni*** **intensity (log epg)**				
*S. mansoni* intensities	0.1076	0.0416	0.026 – 0.189	0.01**

*SME – small scale business ** significant factors associated with left liver lobe hepatomegaly.

R^2^ = 0.0264, Adjusted R^2^ = 0.0140 F = 2.14 and *P*< 0.02.

Linear regression model was constructed for height adjusted ultrasound measurements of the left liver lobe(organ size as continuous variable- i.e. deviations from mean).

Table 6 shows the predictors of height-adjusted ultrasound measurements of the spleen. Spleen measurements were significantly associated with sex of the participants (being male, *P*< 0.002), fishing occupation (*P*< 0.005), village of residence (living in Kayenze village, *P*< 0.039 and Igalagala village, *P*< 0.005), being infected with malaria parasite (*P*< 0.018) and HIV-1 infection (*P*< 0.05).

**Table 6:** **Predictors of spleen size among residents of fishing villages of North-Western Tanzania infected with** ***S. mansoni***

**Variable**	**â**	**SE**	**95% CI**	***P*** **-values**
Sex	0.3968	0.1273	0.14 – 0.65	0.002**
Age	−0.0005	0.0044	−0.009 – 0.0081	0.91
**Occupation**				
SSB*	1			
Peasants	0.2445	0.1966	−0.1413 – 0.63	0.21
Fishing	0.4726	0.2426	−0.003 – 0.94	0.05**
**Villages of residence**				
Igombe	1			
Sangabuye	0.3164	0.1854	−0.047 – 0.68	0.088
Kayenze	0.3333	0.1613	0.016 – 0.65	0.039**
Igalagala	0.7331	0.2630	0.22 – 1.24	0.005**
**Malaria infection**				
No	1			
Yes	0.43	0.1821	0.073 – 0.788	0.018**
**HIV-1 serostatus**				
Negative	1			
Positive	0.5186	0.2645	−0.00076 – 1.037	0.05**
***Schistosoma mansoni*** **intensity (log epg)**				
*S. mansoni* intensities	0.068	0.0381	−0.0061 – 0.1435	0.072

*SSB – small scale business ** significant factors associated with splenomegaly.

R^2^ = 0.0564, Adjusted R^2^ = 0.0445 F = 4.72 and *P*< 0.0001.

Linear regression model was constructed for height adjusted ultrasound measurements of the spleen (organ size as continuous variable-i.e. deviations from mean).

The prevalence of organomegaly (hepatomegaly, splenomegaly and hepatosplenomegaly) in relation to HIV-1 and *S. mansoni* infections is shown in Table 1. Overall, based on the Niamey protocol categories, 13.73% (167/1,216) had an enlarged spleen, 59.70% (726/1,216) had hepatomegaly and 26.56% (323/1.256) had hepatosplenomegaly. There was no significant difference in the prevalence of these morbidities in individuals who were only infected with *S. mansoni* and those who were co-infected with HIV-1/*S. mansoni* (Fisher exact, *P* = 0.40). Similarly, no significant difference was observed in relation to these morbidities in individuals with detectable *S. mansoni* eggs and those with no detectable eggs in their stool samples (*P* = 0.07). To ascertain the importance of demographic factors, HIV-1, malaria infection and intensities of *S. mansoni* infections, on the risk of developing hepatosplenomegaly among individuals infected with *S. mansoni*, a logistic regression model was constructed with hepatosplenomegaly as the outcome variable (Table 7). At bivariate analysis, variables significantly associated with hepatosplenomegaly were village of residence and heavy intensity of *S. mansoni* infection. At multivariable analysis, village of residence (living at Sangabuye village, AOR = 2.21, 95% CI; 1.15– 4.25, *P*< 0.017, Kayenze village, AOR = 2.06, 95% CI; 1.15-3.71, *P*< 0.016) and Igalagala, AOR = 2.62, 95% CI; 1.15-5.93, *P*< 0.021) and being heavily infected with *S. mansoni* infection (AOR = 1.54, 95% CI; 1.01- 2.39, *P*< 0.047) remained independently associated with hepatosplenomegaly. HIV-1 infection was not a predictor (Table 7).

**Table 7:** **Predictors of hepatosplenomegaly among residents of fishing villages of North-Western Tanzania infected with** ***S. mansoni*** **infection**

**Variable**	**COR**	**95% CI**	***P*** **-values**	**AOR**	**95%CI**	***P*** **-values**
**Sex**						
Female	1			1		
Male	1.24	0.89 – 1.71	0.19	1.18	0.79 – 1.76	0.42
**Age (in years)**						
21 – 30	0.81	0.45 – 1.46	0.48	0.65	0.34 – 1.22	0.18
31 – 40	0.81	0.44 – 1.48	0.49	0.59	0.31 – 1.13	0.11
41 – 50	0.91	0.44 – 1.88	0.79	0.66	0.30 – 1.45	0.30
51 - 60	1			1		
**Occupation**						
SSB*	1			1		
Peasants	1.14	0.66 – 1.95	0.64	0.78	0.42 – 1.45	0.43
Fishing	1.51	0.80 – 2.83	0.20	1.12	0.53 – 2.35	0.77
**Village of residence**						
Igombe	1			1		
Sangabuye	1.98	1.15 – 3.43	0.014	2.21	1.15 – 4.25	0.017**
Kayenze	1.84	1.11 – 3.03	0.017	2.06	1.15 – 3.71	0.016**
Igalagala	2.36	1.33 – 4.17	0.003	2.62	1.15 – 5.93	0.021**
**Malaria infection**						
No	1			1		
Yes	1.31	0.78 – 2.16	0.29	1.34	0.78 – 2.28	0.29
**HIV-1 serostatus**						
Negative	1			1	1	
Positive	1.60	0.83 – 3.09	0.16	1.63	0.65 – 1.72	0.82
**Intensity of** ***Schistosoma mansoni*** **infection (epg)**						
1-100 epg	1			1		
101 – 399 epg	1.15	0.75 – 1.76	0.53	1.06	0.65 – 1.72	0.82
≥400 epg	1.59	1.09 – 2.32	0.015	1.54	1.01 – 2.39	0.047**

cOR = Crude Odd Ratio, aOR = Adjusted Odd Ratio.

**Significant P-values for factors associated with hepatosplenomegaly.

## Discussion

In the present study population, we observed a considerably lower prevalence of PPF (grade C-F) than what previously reported in studies from the same geographical setting (16.7%) and on the islands of Ukerewe (36% and 41.5%), North-Western Tanzania [42,43]. Similarly, in comparison to other studies that used the Niamey protocol in sub-Saharan Africa, our prevalence of PPF was low [18,19,44]. The observed variations in PPF between various epidemiological settings can partly be explained by differences in prevalence and intensities of *S. mansoni* infection, the focal nature of transmission of the disease [18,19,45-48], the genetic background of the endemic communities and the length of time individuals have been exposed to *S. mansoni* infection [21,44]. In addition, co-infection with other tropical diseases such as malaria [46], differences in parasites strains [49] and immune responses [5] may contribute to the observed variation in prevalence of PPF between communities.

It has been hypothesized that HIV-1 infection, pathogenesis of which leads to a decreased level of CD4^+^ cell counts [50] may affect the development of hepatic morbidities in HIV-1/*S. mansoni* infected individuals. Thus, individuals co-infected with HIV-1/*S. mansoni* may present with severe PPF grades. We have observed HIV-1 positive individuals either with *S. mansoni* detectable egg in their stool samples or not presenting with PPF. There was no significant difference in prevalence of PPF between individuals co-infected with HIV/*S. mansoni* and those who were not infected with HIV-1 infection. Consistent findings were reported among Kenyan patients co-infected with HIV-1/*S. mansoni* [33]. HIV-1 infected individuals were not at a higher risk of either experiencing severe or different PPF grades associated with *S. mansoni* infection than HIV-1 non-infected individuals [33].

On the other hand, the explanatory factors observed to be associated with PPF in the present study have also been reported by similar studies in *S. mansoni* endemic areas [18,19,21,43]. The gender related association with PPF mainly reflect either gender-specific factors [18,21] or differences in the length of exposure between sexes to risk areas [21]. The development of PPF and its associated morbidities detected by ultrasound, can take years of infection to become manifest. Increased duration of residence in high risk areas, which defines the length of exposure to potential risk areas characterized by an intense transmission and the interactions of immune system and *S. mansoni* eggs trapped in the host body, remains an important factor for the development of these morbidities [5,8,19]. Our present work and those of other authors [19,21], we observed that the risk of periportal fibrosis was higher after an individual have been exposed for more than a decade.

HIV-1 infection or its immunodeficiency, as measured in term of CD4^+^ cell counts, was not associated with PPF. It is worth to note that the presence of relatively non-severely CD4^+^ depleted individuals observed in the present study could have contributed to the lack of association between PPF and HIV-1 or CD4^+^ count levels. However, in the absence of HIV-1 infection, decreased CD4^+^ cell counts with increased PPF grades score have been observed, in individuals who had PPF grades D/E and had lower median CD4^+^ cell counts [33]. Retesting this hypothesis with a larger study population would be needed to allow a definitive conclusion.

In the present study, we also assessed if HIV-1 infection had an influence on the height adjusted size of the left liver lobe, spleen and portal vein diameter among individuals infected with *S. mansoni*. Our findings indicate that the height-adjusted size of the left liver lobe was significantly enlarged in individuals co-infected with HIV-1 and *S. mansoni* compared to individuals infected with *S. mansoni* only. The chronic immune activation associated with the pathogenesis of HIV-1 infection, which also targets other immune cells such as macrophages, does contribute to the increase in the size of the left liver lobe [51,52]. Also, evidence from immunological studies has shown that the left liver lobe predominantly increases in size in *S. mansoni* infection [5,9], and epidemiological studies have shown the relationship between the increase in size of the left liver lobe and the intensity of *S. mansoni* infection [16,18,20,42,53,54]. Perhaps, these observations suggest that, in co-infected individuals, HIV-1 and *S. mansoni* may have additive effects on the size of the left liver lobe, with *S. mansoni* infection influencing the size of the organ in intensity-dependent manner. This observation has also been reported in individuals co-infected with *P. falciparum* and *S. mansoni*, in which *P. falciparum* was associated with hepatosplenomegaly exacerbated by the intensity of *S. mansoni* infection in dependent manner [23,24]. The increase in size of the left liver lobe in HIV-1 infected individuals co-infected with other tropical diseases, pathogenesis of which involve the liver has been also reported for HIV-1/Hepatitis-C co-infection [55]. Our findings on the contribution of *S. mansoni* and HIV-1 infections on the size of the liver present an important clinical feature for evaluating and managing hepatomegaly in HIV-1 infected individuals living in *S. mansoni* endemic countries. *S. mansoni* should be considered as one of the differential diagnosis in causing hepatomegaly in HIV-1 infected individuals.

`In the present study population, no correlation was observed between the left liver lobe size and CD4^+^ T- cell counts among individuals co-infected with *S. mansoni* and HIV-1 infection. Evidence from *S. mansoni* animal model studies, *S. mansoni* infected immunodeficient mice have reduced ability to form protective granulomas and suffer severe hepatic parenchymal cell damage caused by hepatotoxins secreted from parasite eggs [3,56]. In Western Kenya, the level of glutamic oxaloacetic transminase (GOT) enzymes, markers of liver parenchyma damage, did not correlate significantly with CD4^+^ T-cell counts [33], suggesting the reduced level CD4^+^ cells observed were still protective against *S. mansoni* egg hepatotoxins, perhaps by continuing to support the production of anti-egg hepatotoxins. It is worth noting that immuno-suppressed mice studies result a much greater depletion of CD4^+^ T-cells than is seen in the individuals included in this or previous human studies.

On the other hand, *S. mansoni* egg counts correlated significantly with the height-adjusted ultrasound measurements of the left liver lobe and the spleen. In addition, linear regression analysis revealed a number of factors which were either associated height-adjusted ultrasound measurements of the left liver lobe or the spleen. These explanatory factors observed in the present study have also been described in previous studies to interact in causing *S. mansoni*-related left liver lobe hepatomegaly, splenomegaly and hepatosplenomegaly in different age groups [18,20,21]. The association between these factors with organomegaly reflects the variation in transmission intensity of *S. mansoni* between geographical areas, age and sex differences in the exposure to *S. mansoni* risk areas [53]. In high endemic *S. mansoni* transmission areas, children are often infected within the first two or three of life males are suffer high infection intensities than females and intensity of infection increases with age [5]. In these areas, the majority of adults with an increased risk of exposure to infection, such as fishermen, can have significantly enlarged left liver lobe, spleen or both. However, enlarged left liver lobe and spleen, can also found in some areas to be highly prevalent in younger age groups, [5,53]. In these younger groups, left liver lobe size has been shown to correlate with high intensities of *S. mansoni* infection [16,20,53,57]. Similarly, the contribution of *P. falciparum* infection to the left liver lobe and spleen enlargement, and hepatosplenomegaly has been repeatedly reported in endemic areas [23,24]. Where co-infection of *S. mansoni* and *P. falciparum* occurs, these infections have synergistic effects in causing the enlargement of the left liver lobe [23,24].

Our study was subject to some limitations. The small number of *S. mansoni*/HIV-1 co-infected individuals could have affected the power of the study to detect particular changes in those individuals. Thus, some caution must be taken when the interpretation of these findings is carried out. However, despite this limitation, this was a community-based study which recruited a large number of participants living in potential risk areas for *S. mansoni* and HIV-1 infections.

## Conclusion

*Schistosoma mansoni*-related morbidities such as PPF, enlarged left liver lobe, enlarged spleen and hepatosplenomegaly are present in the study population and are associated with various risk factors, including the intensity of *S. mansoni* infection. HIV-1 infected individuals, either with detectable *S. mansoni* eggs in their stool samples or not, had PPF, enlarged left liver lobe, enlarged spleen and hepatosplenomegaly. The PPF grades observed in HIV-1/*S. mansoni* co-infected individuals were similar to those individuals infected only with *S. mansoni*. However, HIV-1 infection or CD4^+^ cell counts were not associated with PPF grades.

Our results indicate that the sizes of the left liver lobe, spleen and portal vein diameter did not vary between individuals infected or not infected with *S. mansoni* infection, thus indicating that current *S. mansoni* infection was not the only underlying cause of enlarged liver and spleen in the present study population. Individuals co-infected with *S. mansoni* and HIV-1 infection had a significantly larger size of the left liver lobe as compared to individuals with *S. mansoni-only* infection, thus indicating that, the two infections could interact in causing the enlargement of the left liver lobe. However, on a regression model, HIV-1 infection was not a predictor of the enlargement of the left liver lobe and immunodeficiencies effects of HIV-1 infection, as measured by CD4^+^ T-cell levels in co-infected individuals, did not correlate positively with the height-adjusted ultrasound measurements of the left liver lobe. Lastly, the implication of our observations is that individuals co-infected with HIV-1/*S. mansoni,* although affected by a *S. mansoni* related organomegaly similar to *S. mansoni* only-infected individuals, could be suffering severe enlargement of these organs. To reduce the burden of other infections and their associated morbidities, they should be either routinely screened and treated when visiting care and treatment clinics (CTC) or other public health measures should be included in the CTC package to reduce their exposure to *S. mansoni* infection.
